# Erythroblastopénie induite par la grossesse: à propos d'un cas et revue de la literature

**DOI:** 10.11604/pamj.2016.24.149.8966

**Published:** 2016-06-21

**Authors:** Drissi Jihad, Kouach Jaouad, Moussaoui Driss, Dehayni Mohamed

**Affiliations:** 1 Service de Gynécologie-obstétrique, Hôpital Militaire d'Instruction Mohammed V, Rabat, Maroc

**Keywords:** Erythroblastopénie, grossesse, transfusions, Erythroblastopenia, pregnancy, transfusions

## Abstract

L’érythroblastopénie induite par la grossesse est une entité pathologique exceptionnelle dont seuls quelques cas isolés ont été décrits dans la littérature anglaise. L'objectif de ce travail est d'analyser les caractéristiques de cette pathologie extrêmement rare à travers la description d'un nouveau cas d’érythroblastopénie induite par la grossesse, et à travers l’étude des 17 cas antérieurement rapportés.

## Abstract (en)

Pregnancy induced erythroblastopenia is an exceptional pathologic entity. Only isolated cases have been described in English literature. The aim of this study is to analyze the characteristics of this extremely rare disease through the description of a new case of pregnancy induced erythroblastopenia and through the study of the 17 cases reported before.

## Introduction

L'anémie au cours de la grossesse est une situation pathologique rencontrée en pratique courante avec comme principales étiologies: l'hémodilution, la carence martiale et/ou en acide folique [1]. L’érythroblastopénie (EBP) reste une cause exceptionnelle. En effet, entre l'origine toxique, virale, auto-immune, lymphoproliférative, myélodysplasique…, la grossesse est une cause rare dont seul quelques cas isolés ont été rapportés dans la littérature anglaise. Depuis la publication de Choudry et al. ayant repris les 13 cas publiés jusqu’à 2007 [2], Andrew C. et Aggrawal S. ont recensés deux nouveaux cas publiés respectivement en 2008 et 2013 [1, 3]. L'objectif de ce travail est d'analyser les caractéristiques de cette pathologie extrêmement rare à travers la description d'un nouveau cas d'EBP induite par la grossesse, et à travers l’étude des 17 cas antérieurement rapportés.

## Patient et observation

Outre notre cas, un total de 17 cas concernant 12 femmes ont été rapportés dans la littérature. Nous avons repris tous les cas publiés pour tenter de préciser les caractéristiques de cette entité pathologique qui, malgré son caractère exceptionnel, ne doit pas être méconnue. Nous rapportons le cas d'une patiente de 27 ans, troisième geste, nullipare, ayant comme antécédents pathologiques: deux avortements spontanés précoces. Ne prenant pas de médicaments au long cours. Ayant consulté pour anémie normocytaire normochrome à 8.8g/dl découverte sur le bilan prénatale réalisé à 14 semaines d'aménorhée (SA), anémie bien tolérée sur le plan clinique, et sans notion de saignement extériorisé. L'examen clinique trouve une patiente en bon état général, stable sur le plan hémodynamique, eupnéique au repos, apyrétique, aux conjonctives légèrement décolorées, aux aires ganglionnaires libres, sans splénomégalie, l'examen cutanéo-muqueux et ostéo-articulaire étaient sans anomalies. Suite à cette découverte fortuite, un bilan étiologique a été demandé objectivant une anémie isolée normocytaire, normochrome: VGM: 82fl, CCMH: 32%, arégénérative avec un taux de réticulocytes effondré à 12000/mm^3^. Les autres lignés leucocytaire et plaquettaire étaient dans les normes. L'enzymologie hépatique, la fonction rénale, le bilan martial et le dosage vitaminique (acide folique et cobalamine) étaient sans anomalies, bilan thyroidien correct. L’étude du frottis de la ponction médullaire a trouvé une moelle riche, avec une lignée érythroide presque inexistante: inférieure à 1%, les autres lignées granuleuses et mégacaryocytaire étaient normalement représentées. Le diagnostic d'EBP a été ainsi retenu. Par ailleurs, les sérologies virales: parvovirus B19, VHB, VHC, VIH étaient négatives. L'IRM médiastinale n'a pas trouvé de thymomes. L’électrophorèse des protéines sériques était normale. L’étude cytogénétique du prélèvement médullaire n'a pas montré d'anomalies en faveur d'une myélodysplasie. Au bilan immunologique: les anticorps antinucléaire et anti ADN natifs étaient négatifs.

Par ailleurs, l’évolution était marquée par l'aggravation rapide clinico-biologique de l'anémie: installation d'une asthénie avec dyspnée stade III de la NYHA, décoloration des conjonctives avec pâleur profonde, tachycardie et une baisse rapide du taux d'hémoglobine (Hb) à 5g/dl en deux semaines. Devant ce contexte aigu un bilan d'hémolyse avec test de Coombs direct, électrophorèse de l'hémoglobine et échographie abdominale à la recherche d'une splénomégalie ont été réalisés revenus sans anomalies. La patiente a été transfusée de 04 culots globulaires(CGR). Aucune complication obstétricale n'a été relevée: pas de contractions utérines ni de métrorragies, ni de modifications cervicales, et à l’échographie il s'agissait d'une grossesse monofœtale évolutive avec une biométrie correspondant à l’âge gestationnel. A sa sortie le taux d'Hb était à 9g/dl. Vu les effets secondaires de la corticothérapie et la contre-indication des immunosuppresseurs, les immunoglobulines intra-veineuses ont été jugés être la thérapie de choix dans ce contexte, mais s'est révélée infructueuse avec une demande transfusionnelle accrue: 2CGR/1-2 semaines en vue de maintenir un taux d'hémoglobine entre 8-10g/dl évitant ainsi le retentissement obstétrical (risque d'avortement, d'accouchement prématuré, de souffrance fœtale chronique, retard de croissance intra-utérin, de souffrance fœtale aiguë et mort fœtale intra-utérine). La patiente a reçu un total de 18 CGR jusqu’à 28 SA, début du troisième trimestre, où est survenue une nette amélioration spontanée: stabilisation du taux d'hémoglobine entre 9-10 g/dl. L'accouchement s'est déroulé par voie basse à terme (39 SA) donnant naissance à un nouveau-né de sexe féminin, avec un poids de naissance de 4 kg. Et un score d'Apgar à 10/10. En post-partum aucun support transfusionnel n'a été nécessaire, et, l'hémoglobine est resté supérieure à 10g/dl jusqu’à complète régression de l'anémie 14 semaines après l'accouchement permettant ainsi de confirmer le diagnostic d'EBP induitepar la grossesse avec, cependant, un haut risque de récidive si une nouvelle grossesse venait à être envisagée. Par ailleurs, il n'y a pas eu d'hémochromatose secondaire aux transfusions. Etude comparative des cas rapportés dans la littérature: un total de 17 cas concernant 12 femmes ont été recensés à travers des recherches effectuées sur Pub Med (1991-2013). Seuls les cas d′EBP induite par a grossesse ont été inclus, les EBP préexistantes ou persistantes après l′accouchement d′origine autre que gestationnelle ont été exclues. Les différents cas ont été résumés dans le Tableau 1.

**Tableau 1:** Cas d’érythroblastopénie induite par la grossesse

Auteur	Age_*_(an)	AG (SA)	Hb (g/dl)	Prise en charge	Issu de la grossesse	Etat du nouveau-né	Délai de rétablissement en PP en semaines
Aggio	15	24	5,2	transfusions	AUH à 30 SA	décès pour prématurité	4
Miyosih	23	12	2,9	transfusions/corticothérapie	AVS à 12SA	_	8
Lehman	23	24	9	transfusions/fer, folate, pyridoxine	accouchement à 40SA	N	6
Majer	18	20	8,3	fer	AVB à terme	décès néonatal	12
Majer	26	28	7,6	transfusions/fer, folate	accouchement à terme	N	4
Bambery	35	T3	?	transfusions/corticothérapie/folate, vitB12	accouchement à terme	décès néonatal	?
Itoh	33	30	2,8	transfusions/corticothérapie	accouchement à 38SA	N	20
Baker	31	10	7,6	transfusions	AVH à 36SA	N	4
Makino	31	19	8,5	transfusions/corticothérapie/fer	AVB à 37SA	N	2
Wakabayashi	28	T3	?	transfusions	accouchement prématuré	N	?
Wakabayashi	31	T2	4,1	transfusions/corticothérapie	accouchement prématuré à 27SA	décès pour prématurité	?
Choudry	33	17	5,1	transfusions/corticothérapie	AVH à terme	N	10
Choudry	36	progestérone	5,3	transfusions/cyclosporine	_	_	28
Choudry	40	14	6	transfusions	AVH à terme	N	8
Andrew	?	?	3.2	transfusions	AVB à terme	N	_
Andrew	30	T1	4.8	transfusions	AVB induit à 36SA	N	12
Andrew	32	15	5.1	transfusions	?	?	?
Aggarwal	28	2 semaines PP	3.2	transfusions	_	_	6
Drissi	27	14	8.8	transfusions/Ig IV	AVB à 39SA	N	14

Age: âge de la patiente; SA: semaine d'aménorhée; Hb: hémoglobin; PP: post-partum; AVH: accouchement par voie haute; AVS: avortement spontané; AVB: accouchement par voie basse; N: normal; Ag: âge gestationnel

En observant les données rapportées sur le tableau, on constate que: cette pathologie concerne les femmes de tout âge avec des extrêmes de 15 à 40 ans et une moyenne de 29 ans; elle peut survenir à tout âge gestationnel aussi bien au 1^er^, 2^ème^ ou 3^ème^ trimestre, le diagnostic peut même être posé en post-partum (cas rapporté par Aggarwal); dans plus de 70% des cas, le diagnostic est posé devant une anémie sévère (Hb<8 g/dl); les transfusions itératives ont constituées la base de la prise en charge de ces patientes (exception faite pour le premier cas décrit par Majer où la prise en charge a consisté uniquement à une supplémentation martiale). 6 cas (soit 33% des femmes) ont bénéficié d′une cortiocothérapie à base prednisolone mais sans efficacité constatée par rapport au groupe traité par transfusions seules. Dans notre cas l′administration d′immunoglobulines polyvalentes s'est avérée inefficace; le risque de récidive au cours des grossesses chez ces femmes est illustré par les cas rapportés par Choudry et Andrew, avec probablement un effet inducteur de la progestérone; que l′accouchement se soit déroulé par voie basse ou césarienne, la majorité des grossesses ont eu une issue favorable avec des nouveau-nés en bonne santé, les quelques décès notés ont été inhérents à la prématurité; la régression rapide de l′anémie après vacuité utérine était de règle, avec un délai variant de 2 à 20 semaines et une moyenne de 10 semaines.

## Discussion

L′EBP est une cause rare d′anémie. D′origine centrale, elle est caractérisée par l′extinction isolée de la lignée érythroblastique. Le diagnostic doit être évoqué devant toute anémie normochrome normocytaire, parfois macrocytaire, isolé; sans anomalie plaquettaire ou leucocytaire, arégénérative. Le diagnostic positif repose sur l′étude du frottis médullaire qui relève une franche hypoplasie de la lignée érythroïde coexistant avec une cellularité normale des autres lignées. La biopsie ostéomédullaire, geste invasif, n′est pas nécessaire pour établir le diagnostic [1–5]. Les étiologies des EBP acquises sont multiples. Entre l'origine toxique, virale, auto-immune, lymphoproliférative, myélodysplasique; la grossesse demeure une cause exceptionnelle à évoquer devant toute anémie normochrome normocytaire découverte pour la première fois au cours de la grossesse et dont le bilan étiologique s′avère négatif. Cependant, la confirmation se fera souvent à postériori devant la régression spontanée de l′anémie dans les quelques semaines qui suivent l′accouchement [3]. La physiopathologie de l′EBP induite par la grossesse reste mal élucidée. Les 3 études expérimentales réalisées in vitro par Wakayashi et Takaku, Itoh et al., Baker et al. suggèrent l′implication d′un mécanisme auto-immum: un anticorps antiérythroïde (anti BFU-E), dont la production serait déclenchée par les antigènes foetaux d′origine paternelle, est incriminé dans la pathogénèse de la maladie. Quoiqu′il soit le type IgG, cet anticorps ne semble pas traverser la barrière placentaire et semble donc être sans effet délétère sur le foetus et le nouveau-né [1, 2]. Choudry et al. a soulevé la possible implication des changements hormonaux survenant pendant la grossesse dans le déclenchement du processus auto-immun chez les femmes prédisposées, et ceux, devant la constatation d′un cas d'EBP provoquée par la prise de medroxyprogestérone chez une femme ayant une histoire de deux épisodes d′EBP induites par la grossesse. Des études expérimentales approfondies devront être réalisées dans ce sens [2]. La prise en charge repose essentiellement sur les transfusions itératives en vue de maintenir un taux d′hémoglobine compris entre 8 et 10 g/dl évitant ainsi les complications obstétricales et le retentissement fœtal, parfois fatal, de l′anémie [3].

Certains des quelques auteurs qui ont été confrontés à ce type de pathologie ont associé aux transfusions la corticothérapie à base de prédnisolone mais sans net effet bénéfique constaté, cela amène à dire que cette thérapeutique devrait être évitée dans ce cas particulier d′EBP induite par la grossesse [1–3]. Mant MJ. a rapporté un cas d′EBP chronique idiopathique qui a bien évolué durant la grossesse sans aucun besoin transfusionnel sous immunoglobulines intraveineuses; faisant de cette thérapeutique une option prometteuse dans la prise en charge des EBP induite par la grossesse [3, 6]. Toutefois, ce choix a été sans succès chez notre patiente. A noter qu′il n′y a pas lieu d′indiquer l′interruption de la grossesse [2]. La surveillance doit se faire en collaboration entre obstétricien et hématologue. La surveillance hématologique se base sur la surveillance régulière du taux d′hémoglobine. La surveillance obstétricale, à l′instar de toute grossesse à haut risque, doit être rapprochée particulièrement au troisième trimestre. Pour ce qui est du mode d′accouchement, il sera dicté par les conditions obstétricales en veillant à minimiser les pertes sanguines au cours de l′accouchement. Au total, une bonne gestion de l′anémie est garante d′un bon déroulement de la grossesse. Contrairement aux autres types d′EBP, celle induite par la grossesse garde un bon pronostique devant le prompt rétablissement dans les semaines qui suivent l′accouchement, avec cependant un haut risque de récidive au cours des grossesses ultérieures justifiant une surveillance rigoureuse si une nouvelle grossesse venait à être envisagée. Par ailleurs, tout traitement à base de progestatifs, s′il ne peut être évité, devra être administré avec parcimonie chez ces patientes [2].

## Conclusion

L'EBP induite par la grossesse est une entité pathologique exceptionnelle à évoquer devant toute anémie normocytaire normochrome arégénérative découverte au cours de la grossesse. La prise en charge repose sur les transfusions itératives, il n'y a pas lieu d'interrompre la grossesse, la corticothérapie devra être évitée dans ce contexte. Une bonne gestion de l'anémie est garante d'un bon déroulement de la grossesse sans conséquences fœtale ou néonatale. Le pronostique est bon devant la prompte guérison en post-partum avec cependant un risque de récidive au cours des grossesses ultérieures. Tout traitement à base de progestatifs devra être évité chez ces patients.
